# Associations between social networks, cognitive function, and quality of life among older adults in long-term care

**DOI:** 10.1186/s12877-024-04794-9

**Published:** 2024-03-04

**Authors:** Laura Dodds, Carol Brayne, Joyce Siette

**Affiliations:** 1 The MARCS Institute for Brain, Behaviour and Development, Western Sydney University, Westmead, NSW Australia; 2 Cambridge Public Health, Cambridge University, Cambridge, UK

**Keywords:** Aged care service utilisation, Social networks, Cognitive performance, Quality of life, Nursing home

## Abstract

**Background:**

Having rich social networks is associated with better physical and cognitive health, however older adults entering long-term care may experience an increased risk of social isolation and consequent negative impacts on cognitive function. Our study aimed to identify if there is an association between accessing specific types of services or activities within long-term care on social networks and cognition.

**Methods:**

A cross-sectional study of 96 residents from 2 aged care providers in New South Wales, Australia. Residents were given a battery of assessments measuring social network structure (Lubben Social Network Scale, LSNS-12), quality of life (EuroQol 5D, Eq. 5D5L) and cognitive function (Montreal Cognitive Assessment, MoCA). Demographic factors and service use factors were also collected from aged care providers’ electronic records. Independent sample t-test, ANOVA and linear regression analyses were used to explore associated factors for cognition.

**Results:**

Residents had a mean age of 82.7 ± 9.4 years (median = 81) and 64.6% were women. Most residents had cognitive impairment (70.8%) and reported moderate sized social networks (26.7/60) (Lubben Social Network Scale, LSNS-12). Residents who had larger social networks of both family and friends had significantly better cognitive performance. Service type and frequency of attendance were not associated with cognitive function.

**Conclusions:**

Among individuals most at risk of social isolation, having supportive and fulfilling social networks was associated with preserved cognitive function. The relationship between service provision and social interactions that offer psychosocial support within long-term facilities and its impact over time on cognitive function requires further exploration.

## Introduction

As we approach older adulthood, opportunities for social interaction, availability of social support and active participation in socially stimulating activities often decrease. However, the value assigned to psychosocial components of ageing such as contribution to community and embodiment of social roles are identified as hallmarks of successful ageing amongst older adults [1]. Thus, maintaining social engagement is not only important throughout the life course it is particularly valuable as we get older. Remaining socially connected has beneficial effects on our physical and mental health, life satisfaction, quality of life, inclination to adopt positive health behaviours and reduced risk of functional decline and mortality [2]. The protective effects of sustaining social engagement on physiological processes associated with the delay of cognitive decline and dementia suggest it may support inflammatory processes, cardiovascular health and cognitive reserve, which refers to the underlying processes that equip the brain with flexibility and capacity to function adequately despite brain changes or damage [3–7].

A larger evidence base for structural (i.e., network size, frequency of contact and social activity participation) [6, 8] compared to functional aspects (i.e., social support and satisfaction with network) [9–11] of social relationships have been associated with a reduced risk of developing dementia. In an ageing society, without a cure yet, and with prevalence rates remaining high and rising rapidly in certain regions, the impact of dementia will continue to place large financial pressure on global health care systems through recurrent and prolonged demand for both health and aged care services [12–14]. Thus, deconstructing key elements of social stimulation and determining associations with cognition are worth exploration.

Recent cross-sectional and longitudinal studies provide further insight into the mechanisms underlying the relationship between social connection and cognition in older adults. Cross-sectional studies highlight that having larger social networks [15], more frequent interaction with friends rather than family [16], and receiving adequate social support [17] are associated with better cognitive function. Additional studies have found that better cognition is linked with older adults’ ability to maintain and create connections between their network members [18].

Longitudinal studies reinforce similar results. Greater emotional support [11], and larger networks consisting specifically of friendships [19, 20] are associated with sustained levels of overall cognition [21]; and that social disengagement [22, 23] and infrequent social activity participation is associated with an increased chance of developing dementia [24]. Furthermore, it has been suggested that social contact helps to build and sustain cognitive reserve which can act as a protective factor for future cognitive decline [25].

However, synthesised findings from systematic reviews and meta-analyses collectively demonstrate inconsistent evidence for satisfaction with social networks and that poor social engagement, defined by living alone, inadequate social support, minimal social contacts and lack of social activity is associated with increased dementia risk in later life [8–10].

The above-mentioned studies are primarily based on older adults living in the community or retirement villages and cannot be generalised to older adults requiring long-term care in permanent residential care homes, defined as care required in multiple facets of living for a prolonged period [26]. The social environment within institutionalised settings is often distinct from the general population and distinguished by substantial decrease in the quantity and quality of their social connections [27–29]. Typically, residents have significantly fewer social ties, a low number of new connections being made and of these, a lack of mutual friendships or reciprocity [29–31]. Residents also rely on pre-existing relationships with family and friends outside of the facility, are often unable to form meaningful social connections with others within the facility due to physical limitations, cognitive and communication difficulties, and have less opportune moments for social activity due to restrictive routines [31].

In a systematic review of factors associated older adults’ social networks, nine studies in institutional care were identified [30]. Of these, having more connections with others indicated higher quality of life and cognitive status [32] and similarly, residents with higher cognitive deficits also reported less social ties [28]. However, several limitations prompt the need for further evaluation of the social factors for cognition in long-term care. These include inconsistently administered social network tools [28, 29, 32, 33], small sample sizes (ranging from 10 to 36 participants), and data obtained from only one aged care provider [28, 29, 32, 34], which all limit generalisability and interpretation.

Long-term care psychosocial services and activities (e.g., exercise class, reminiscence group) are primary sources of social interaction for residents in aged care facilities. However, the investigation of service use factors including type and frequency of activities and their impact on cognitive function inside facilities remains unknown [7]. Current knowledge is derived from activity participation amongst community dwelling older adults (e.g., facilitator led discussions, field trips, outings, religious activities, amongst others) which has propitious outcomes for better brain health and cognitive functioning [7, 35–37]. For example, by participating in a larger variety of services or activities [7, 37], older adults in the community have the potential to expand their social interactions and gain educational and creative opportunities, which may assist in maintaining quality of life [38, 39], improve functional health outcomes (activities of daily living) [40] and preserve cognitive performance [37]. Yet, this direct association remains unexplored. More extensive analysis of activity participation and their association with cognitive function in long-term care may further assist with optimising resident outcomes. During the pandemic, lockdowns posed a significant challenge for maintaining social contact for older adults due to their vulnerability to the disease and increased physical isolation [41]. Evidence suggests that being able to maintain a feeling of connection with a community may have had protective effects on perceived social isolation during the restrictive period of COVID-19 amongst community-dwelling adults, despite marked reductions in quality of life, wellbeing and life satisfaction [42, 43].

Our study thus aimed to identify the association between accessing specific types of services within long-term care, social networks and cognition pre-pandemic. This research will further assist in understanding the extent of the detrimental impact of the pandemic on social networks and cognition in aged care facilities faced with staffing restrictions, changes to usual visiting practices and a halt in psychosocial and recreational activities.

## Methods

### Study design

A cross-sectional study design in seven facilities across two aged care providers comprising both metropolitan and regional-based facilities in the Central Coast and Greater Sydney regions of New South Wales, Australia was conducted. In total, the facilities housed 635 residents. A complete sampling approach was used, where residents who satisfied the inclusion and exclusion criteria (i.e. aged 55 years or older, no diagnosis of advanced dementia, no history of significant brain trauma, stroke or epilepsy, no vision or severe hearing impairment) were invited to participate by their facility manager. Residents who expressed interest were then approached by members of the research team. Data was collected between September 2019 and January 2020.

### Ethical considerations

The Macquarie University Human Research Ethics Committee provided ethical approval for this study prior to commencement of participant recruitment (reference number: 5159).

### Data collection and procedure

Participants were assessed on competency to provide consent per the study’s ethics protocol prior to obtaining written consent and engagement in the interview. Interviews ranged from 20 to 40 min in duration. Each participant provided information about their demographics (age, gender, marital status, educational level) and completed validated measures on social networks, cognition, and quality of life. Participants were then provided with a gift voucher to acknowledge their time and input into the research project. Following the completion of interviews, service use and resident information in the month prior to the date of interview assessment was extracted from the providers’ electronic database management system.

### Measures

#### Demographics

Demographic information was collected from the individual including age, gender, relationship and pension status, country of birth and main language spoken, highest level of education attained, religious affiliation, previous occupation, medical conditions/history (including number of falls in past 6 months) and current medications. Participant age, marital and pension status, medical conditions/history (including falls and mobility status), and current medications were verified with electronic service provider data.

#### Social networks

Social networks were assessed using the Lubben Social Network Scale-12 (LSNS-12) [44] which measures structural (e.g., network size, composition), interactional (e.g., duration of respondents’ relationships, frequency of contact, quality of exchange) and functional components (e.g., purpose of support) of the respondent’s contacts. The total score is calculated by summing all the items, with a higher score indicating more social engagement and better networks. For the LSNS-12, the score ranges between 0 and 60. LSNS-12 has strong methodological qualities (internal reliability 0.83) and has been developed specifically for older adults [44, 45].

#### Cognition

Classification of cognitive status was conducted by in-person administration of the Montreal Cognitive Assessment (MoCA) [46], a rapid screening instrument for mild cognitive dysfunction. The MoCA has an estimated duration of ten minutes, and assesses visuospatial/executive function, naming, episodic memory, attention, language, abstraction, and orientation. The total possible score is 30 points. The original manual cites a score of 26 or above as normal, however a cut-off score of 23 has recently been recommended for greater diagnostic accuracy [47]. The MoCA has acceptable psychometric properties [48], and at an optimal cut-off of below 22 for MCI, the MoCA is considered to counteract the limitations of the Mini-Mental State Examination (MMSE), achieving significantly superior values in sensitivity, specificity, positive predictive value, negative predictive value, and classification accuracy in High Income Settings [49]. The MoCA has been validated for a variety of different languages and aetiologies [50–53], including Alzheimer’s disease.

#### Quality of life

Quality of life was measured using the EQ-5D-5L scale, which is a generic instrument consisting of a self-administered health index and a visual analogue scale (VAS), a 20-cm scale in which respondents are asked to rate their current health state [54]. It is a brief instrument, representing five dimensions of health related quality of life [55], as opposed to quality of life in general [56, 57]. The EQ-5D-5L contains five domains: mobility, self-care, pain/discomfort, usual activities and anxiety/depression. There are five levels per dimension: no problems, slight problems, moderate problems, severe problems or extreme problems. For the items measuring experience of pain and anxiety, the five ratings relate to the severity of symptoms. Utility scores quantify health-related quality of life along a continuum that ranges from − 0.59 (worst health) to 1.00 (perfect health). Respondents are asked to mark their current health state on a 100-point VAS scale, with 100 representing the ‘best imaginable health state’ and 0 representing the ‘worst imaginable health state’.

EQ-5D-5L data was converted into health utility scores, providing a single evaluation, using the time trade-off method based on the tariff developed for the EQ-5D-5L index in the UK [58]. This scale has high measurement properties, improved discriminatory power and establishing convergent and known groups validity [58].

### Service use

Frequency of service use was extracted from the providers’ electronic database management system one month prior to the date of interview for each resident. Frequency refers to the number of times a participant attended a particular service type in the last 30 days. A one-month time-frame was selected for numerous reasons. It ensured that the data captured reflected the current state of service use amongst residents and allowed for precision and targeted analysis when evaluating the impact of psychosocial activities which is particularly important when activities vary each month. It also enhanced efficiency in data storage, processing and analysis as well as minimised staff burden without compromising the value of the data. To streamline service types between providers, the research team re-categorised existing data into four distinct categories: (1) *cognitive* (e.g. quizzes, puzzles, crosswords, news and current affairs, cognitive and sensory stimulation, music and memory, dementia specific programs, reminiscence therapy), (2) *physical* (e.g. walks, exercises, fine and gross motor skill activities, chair aerobics, tai chi), (3) *social* (e.g. conversation groups, one on one time, special events, visitors, happy hour, breakfast club, coffee club, school student visit, high tea, men’s club, knitting club) and (4) *personal interests* (e.g. horticulture, spiritual, reading, art and craft, pet therapy, musical, cooking group, outings, hairdresser visits, shopping). Total number of service attendances was also computed.

### Other resident information

Additionally, resident date of admission into facility and medical conditions and mobility status were also extracted from provider’s electronic database management system.

### Control variables

Potential confounding variables of demographic and socioeconomic determinants on cognitive function were controlled for in the analyses and included age, gender, education and marital status as identified from the literature [59]. There were no missing data.

### Statistical analysis

Descriptive analyses for all continuous variables, including variables that were not normally distributed, were reported as mean and standard deviation (SD), and categorical variables were reported as frequencies and percentages. Multiple linear regression analyses were used to determine associations between: (i) social networks and quality of life; (ii) social networks and residential care service utilisation; and (iii) social networks and cognition. Analyses were performed to build a step-wise model adjusted for demographics (age, gender, marital status and education) identified in the literature as potential covariates. Adjusted R^2^ values were reported for regression models to indicate the proportion of variance explained by variables in the model. Regression coefficients were also reported. All analyses were conducted using SPSS V25.

## Results

Descriptive statistics of sociodemographic characteristics are shown in Table 1. Participants (*n* = 96) had a mean age of 82.7 years (median = 81), were mostly women (64.6%), widowed (58.3%), born in an English-speaking country (88.5%), and been a resident of the facility for an average of 2.3 years.

**Table 1:** Demographics and characteristics of 96 older adults in long-term care

Characteristic	All N (%)
**Gender**	
Female	62 (64.6)
Male	34 (35.4)
**Age (Mean [SD], range)**	82.7 [9.4], 60–103
55–64	3 (3.1)
65–74	18 (18.8)
75–84	33 (34.4)
85+	42 (43.8)
**Relationship status**	
Widowed	56 (58.3)
Married	27 (28.1)
Divorced	6 (6.3)
Never married	6 (6.3)
**Country of birth**	
English-speaking country	85 (88.5)
Non-English-speaking country	11 (11.5)
**Receiving pension**	
Yes	90 (93.8)
No	6 (6.3)
**Years of education (Mean [SD])**	6.41 [16.8]
Up to 6 years	11 (11.5)
Up to 10 years	59 (61.5)
Up to 12 years	8 (8.3)
Trade or technical certificate	6 (6.3)
Bachelor’s Degree	6 (6.2)
Postgraduate Degree	3 (3.1)
Unknown	3 (3.1)
**Years in long-term care (Mean, [SD], range)**	2.3 [2.6], 0–20
<1 year	34 (35.4)
1–2 years	22 (22.9)
2–3 years	21 (21.9)
>3 years	19 (19.8)
**Frequency of long-term care activity attendances** ^1^ **(Mean, [SD], range)**	52.6 [44.0], 3–156.4
Cognitive^2^(Mean, [SD], range)	8.8 [10.9], 0–39.5
Physical^3^ (Mean, [SD], range)	10.7 [11.0], 0–36.6
Social^4^ (Mean, [SD], range)	17.2 [12.8], 0–59.0
Personal Interest^5^ (Mean, [SD], range)	15.9 [16.4], 0–57.9
**Attendances at multiple activity types** ^6^ **(Mean, [SD], range)**	3.5 [0.8], 2–4
2^7^	17 (17.7)
3^8^	16 (16.7)
4^9^	63 (65.6)
**Quality of life**	
Equation 5D VAS score (mean, [SD], range)	70.2 [19.7], 10–100
Equation 5D Utility index (mean, [SD], range)	0.6 [0.3], -0.15–1
**Social network score (Mean, [SD], range)**	26.7 [11.2], 0–52
Family network score (Mean, [SD], range)	18.4 [7.6], 0–42
Friends network score (Mean, [SD], range)	9.3 [8.4], 0–26
**Cognition score (Mean MoCA [SD], range)**	16.8 [6.3], 3–27
Cognitive impairment	68 (70.8)
No cognitive impairment	28 (29.2)

^1^Refers to the mean number of total activities residents attended in the month prior to interview assessment

^2^Refers to the mean monthly attendances at cognitive activities or games that involve problem solving, decision making and/or sense making

^3^Refers to the mean monthly attendances at physical activities that involve movement and/or exercise

^4^Refers to the mean monthly attendances at social activities that facilitate connection to peers, groups or the wider community

^5^Refers to the mean monthly attendances at personal Interest or spare-time recreational activities that one chooses to do to because they place value in it or have a particular interest or liking towards

^6^Refers to the mean number of activity types attended in the month prior to interview assessment

^7^Refers to the proportion of residents who attended 2 activity types in the month prior to interview assessment

^8^Refers to the proportion of residents who attended 3 activity types in the month prior to interview assessment

^9^Refers to the proportion of residents who attended 4 activity types in the month prior to interview assessment

Residents reported greater ties with their family circle (18.4 out of 30) more so than friends (9.3 out of 30) which contributed to a mean social network score of 26.7 (out of a possible 60), indicating residents were socially integrated. A total social network score of less than 20 indicates an extremely limited social network, with 29.2% participants meeting this criterion. There was substantial variation (range 0–52, interquartile range [IQR] 35,75).

Quality of life was moderately low with mean utility and EQ-VAS scores of 0.6 (SD = 0.3) and 70.2 (SD = 19.7), respectively. Problems with mobility were the most frequently reported, two-thirds (68.8%) with slight to extreme mobility issues (level 2 or more), and around one in seven (14.6%) reporting that they are unable to walk (level 4 or 5) (Table 2). In contrast nearly three-quarters of residents (74.0%) reported no issues carrying out daily activities. Majority of residents were cognitively impaired with 70.8% of individuals scoring less than 22 and an average MoCA score of 16.8 (SD = 6.3) out of a possible 30. There were no significant associations between quality of life (EQ-VAS score and utility index) and cognition (*p* > 0.05).

**Table 2:** Quality of life responses by domain

	EQ-5D-5L Domains N (%)				
	Mobility	Self-Care	Usual Activities	Pain/ Discomfort	Anxiety/ Depression
**No problems**	30 (31.3)	34 (35.4)	71 (74.0)	48 (50.0)	50 (52.1)
**Slight problems**	31 (32.3)	29 (30.2)	15 (15.6)	27 (28.1)	30 (31.3)
**Moderate problems**	14 (14.6)	17 (17.7)	7 (7.3)	11 (11.5)	12 (12.5)
**Severe problems**	7 (7.3)	13 (13.5)	3 (3.1)	9 (9.4)	4 (4.2)
**Extreme problems**	14 (14.6)	3 (3.1)	0 (0.0)	1 (1.0)	0 (0.0)

Residents attended an average number of 52.6 (SD = 44.0) activities across a one-month period with 63% of residents attending all four types of activities (Table 1). Of these, residents tended to attend social activities the most (mean = 17.2 sessions, SD = 12.8) and cognitive activities the least (mean = 8.8 sessions, SD = 10.9). There was no clear relationship between frequency of long-term care activity attendances or taking part in multiple activity types and cognition.

In the univariate analysis, younger age (*p* = 0.039), more years of education (*p* = 0.002), high social networks (*p* = 0.04), high family network score (*p* = 0.04) and being born in an English-speaking country (*p* = 0.003) were significant predictors of higher cognitive function (Table 3). Linear stepwise regression of cognitive function (model adjusted R^2^ = 0.17, *F*[13,76] = 2.42, *p* = 0.002) using social network, service use, quality of life and demographic parameters resulted in only one significant predictor: residents with high social networks (ß=0.15, p 0.01) had higher cognition. Service use type or frequency did not predict cognition in this model (Table 4).

**Table 3:** Association of sociodemographic and service use factors to cognitive function

Characteristic	Cognitive function Mean MoCA score (SD)	F	p-value
**Gender**		0.37	0.55
Female	17.08 (5.87)		
Male	16.26 (7.05)		
**Age**		**2.91**	0.04*
55–64	13.00 (10.39)		
65–74	20.44 (6.10)		
75–84	15.85 (6.41)		
85+	16.24 (5.57)		
**Relationship status**		0.09	0.99
Widowed	16.68 (6.11)		
Married	17.04 (6.12)		
Divorced	17.83 (7.86)		
Never married	15.83 (9.00)		
**Country of birth**		9.06	<0.001*
English-speaking country	17.46 (6.03)		
Non-English-speaking country	11.64 (6.15)		
**Receiving pension**		1.26	0.26
Yes	16.98 (6.23)		
No	14.00 (7.13)		
**Years of education**		**3.72**	<0.001*
Up to 6 years	15.36 (6.30)		
Up to 10 years	17.86 (5.43)		
Up to 12 years	13.38 (7.01)		
Trade or technical certificate	12.67 (7.39)		
Bachelor’s Degree	18.33 (7.09)		
Postgraduate Degree	24.67 (1.16)		
Unknown	7.33 (3.5)		
**Years in long-term care**		0.66	0.58
<1 year	15.91 (6.51)		
1–2 years	18.27 (6.60)		
2–3 years	17.051 (5.55)		
>3 years	16.37 (6.45)		
**Frequency of long-term care activity attendances**		0.05	0.83
Above the mean	16.90 (6.46)		
Below the mean	17.18 (6.13)		
**Attendances at each activity type**			
Cognitive		0	0.98
Above the mean	17.03 (5.99)		
Below the mean	17.07 (6.45)		
Physical		0.38	0.54
Above the mean	16.33 (6.49)		
Below the mean	17.13 (6.18)		
Social		0.74	0.40
Above the mean	16.485 (6.67)		
Below the mean	17.61 (5.83)		
Personal Interest		0.02	0.90
Above the mean	16.94 (6.51)		
Below the mean	17.12 (8.16)		
**Attendances at multiple activity types**		0.03	0.97
2	16.88 (6.39)		
3	17.13 (6.48)		
4	16.68 (6.31)		
**Quality of Life EQ5D utility index**		0.01	0.93
Above the mean	16.8 (6.61)		
Below the mean	16.9 (5.83)		
**Social networks**			
**Total network score**		**4.47**	0.04*
Above the mean	18.13 (6.44)		
Below the mean	15.46 (5.90)		
**Family network score**		**4.40**	0.04*
Above the mean	18.06 (5.90)		
Below the mean	15.41 (6.48)		
**Friends network score**		1.24	0.27
Above the mean	17.49 (6.30)		
Below the mean	16.06 (6.27)		

*Significant at *p* < 0.05

**Table 4:** Summary of linear regression analysis for predictors of cognitive performance in 90 older adults in long-term care

Predictors	Cognitive function (*N* = 96)			
	B^1^	SE B^2^	ß^3^	p-value
***Constant***	25.16	6.85	-	<0.001
**Age**	− 0.10	0.07	− 0.13	0.18
**Gender**	1.50	1.49	0.12	0.32
**Marital status**	− 0.04	0.06	− 0.07	0.52
**Country of birth**	− 4.02	2.09	− 0.20	0.06
**Education**	− 0.08	0.04	− 0.21	0.06
**Quality of life**	1.99	2.49	0.09	0.43
**Length of Stay**	− 0.40	0.55	− 0.07	0.47
**Social Network Total Score**	0.14	0.06	0.26	0.02*
**Service Type – Cognitive activity**	0.03	0.17	0.04	0.88
**Service Type – Physical activity**	− 0.20	0.12	− 0.36	0.12
**Service Type – Social activity**	0.08	0.06	0.16	0.18
**Service Type – Personal Interest activity**	0.05	0.10	0.13	0.64
**Attendances at multiple activity types**	− 0.50	1.10	− 0.06	0.65
		**R** ^**2**^	**0.17**	
		**F**	**2.42**	

^1^refers to the unstandardized beta (B); ^2^ refers to the standard error for the unstandardized beta (SE B); ^3^ refers to the standardized beta (β); *Significant at *p* < 0.05

## Discussion

We investigated cross-sectional relationships between the characteristics of social networks, cognition, quality of life and service activity frequency and type in the preceding month amongst older adults in two long-term care settings. Individuals with larger social networks including family and friend connections had better cognition. The relationship between social networks and cognitive function were independent of quality of life and specific activity type and attendance. These findings provide useful insights into the factors associated with better cognition in older long-term care adults and contributes to the view that aged care reform may be necessary to optimise activities and opportunities that support residents’ cognition and social wellbeing.

Frequency of attendance and type of scheduled activities within the facility was not related to cognitive performance. Given the promising evidence on the link between service attendance and better outcomes on older adults’ social engagement, brain health, functional status and wellbeing [35, 37] this finding is surprising, but also hopeful post pandemic in that the detrimental impacts of reductions in recreational activities for residents in long-term care may have influenced some aspects of physical and mental health but not others [60]. Although activity attendance is the most consistently reported indicator of social participation across community day centres and long-term care facilities [61], our study highlights that the relationship between activities, social networks and cognition is more complex. It may be that residents with declining cognition and function are less likely to participate in social activities [62], or that existing larger social networks and guaranteed social engagement have a protective effect on cognitive function [5, 6]. However, evidence over time is inconclusive and specific to cognitive domains [7, 35, 37, 63]. The social connections developed in long-term care can overcome social isolation, create a sense of autonomy and counterbalance resident’s physical limitations in some circumstances [64]. Therefore, it is essential to determine whether scheduled activities act as facilitating agents or preclude the broadening of social networks and improvements in cognition as favourable mechanisms remain unknown. Future studies adopting a longitudinal design with longer follow-up periods and a larger sample size may contribute further knowledge.

In other studies, residents have reported that most social interaction within the facility occurs in public spaces e.g., corridor, café or during visits/outings with family members rather than scheduled activities [31, 65, 66]. Planned activities were seen as spaces where residents were physically together but were insufficient at promoting positive participation and conversation e.g. assigned seating at mealtimes [31, 67], thus residents remained unfamiliar with each other [31, 65]. This implies that broadening social networks through traditional service provision may be unattainable. Instead, it calls for research into the structure, quality and nature of engagement in recreational programming in long-term care to provide residents with psychosocial support from meaningful, ecological and fulfilling social encounters.

Having larger overall social networks was the main driving factor for greater cognition. This is consistent with previous cross-sectional [15, 32, 37] and longitudinal data [22–24] for older adults in the general population and those accessing community aged care services. Our results suggest that having access to a greater number of close social ties, and repeated social engagement unique to this setting (i.e., with staff, family members, long-term friends, other residents) may reflect positively on cognitive health. Previous evidence suggests a residents’ typical social network in long-term care is static (no incoming or outgoing social ties), with friendship ties that lack mutuality [30]. Additionally, long-term care characteristics such as facility size, limited choice in social partners, reduced resources for social engagement, unique resident mix (which predominantly consists of individuals who are cognitively impaired) and various other uncertainties (length of time until death, major health deteriorations and discharges) are recognised barriers to forming new friendships [28, 31, 68, 69]. Considering this, and the higher number of family contacts reported in our study, residents may invest a greater degree of effort and emotional affection into maintaining established long-term relationships with family members [31].

Previous research suggests that years of education and actively working to preserve cognition across the life-span provides protective effects from cognitive decline (e.g., helping to build cognitive reserve and delaying the clinical expression of dementia) [36, 70, 71]. Distinct from these studies, this study suggests education was not significantly associated with cognitive impairment. A reason for this may be that in comparison to adults in the general population or accessing community care, individuals in residential aged care are older, with 58% of people in long-term care over the age of 85 compared to 41% of those accessing home care [72]. This is reflected in the average age of our study population which is relatively high (82.7). The protective effects of education attainment may only work below a threshold at which normative age-related impairments may surpass [73]. Therefore, the extent to which years of education can stave off cognitive decline is potentially limited in the later years of life.

### Limitations

This study has several limitations. Firstly, using a cross-sectional design, causality is not assumed as reverse causality is a possibility, with poorer functional and cognitive status being associated with ageing, which can influence the ability to navigate social relationships [18, 62] and lead to social withdrawal [74]. Furthermore, older age, cognitive impairment and social vulnerability are also known predictors of entry into long-term care [75]. It was beyond the scope of this study to identify whether participants had experienced diminishing social networks or some level of cognitive impairment prior to admission into long-term care, however future studies should adopt a longitudinal approach to investigate the relationship between psychosocial service use, cognition, and social networks over longer periods of time.

Secondly, we were only able to recruit from two aged care providers, and the study outcomes and nature of the population required researchers to select participants through the provider. Willingness to participate and eligibility was assessed by the staff, which may be a potential source of bias. The lack of missing data is unusual suggesting some bias in selection of those able and sufficiently well to participate and willing to respond to all questions. Despite this, researchers did not encounter any drop outs when approaching residents to participate, enhancing the generalisability of the findings [76]. Studies should further explore objective measures of quality of life and cognition as reported measures remain high for this population group (i.e., over three quarters reporting no assistance required for activities of daily living), however residents were pre-selected by staff and thus might be biased. Another option would be verifying self-reported responses with staff or family reported observations to ensure researchers are not solely relying on residents’ perceived needs. Another source of bias could stem from the decision to extract one month of psychosocial service use data, whereas a longer period of time would more sufficiently depict typical resident activity attendance. Finally, this study is limited in its assessment of psychosocial service use through activity attendance and excludes those who may have specific social participation goals (i.e. prefer not to participate), and neglects the shared experiences that naturally occur outside of these activities in long-term care [31].

### Implications

The nature and design of activity programs in long-term care could also be a critical factor contributing to cognitive health. Many activities in long-term care are not tailored to the functional or social needs of the residents and are incongruent with their interests and expectations of friendship [29, 61]. For example, residents with cognitive impairment often refrain from attending activities or adopt a passive and superficial approach to interactions due to communication difficulties; residents with functional limitations i.e., visual or hearing impairments may require assistive technologies to participate which are not always accessible [77]; and those requiring more physical care may also have restrictive routines which limit their activity choices [61]. It is possible that inclusion of resident preferences for recreational programming was inadequate, or that activities were unable to provide relief from the monotony of their daily routine [31, 78].

Indeed, recreational activities in long-term care are often void of opportunities for choice and autonomy, learning or personal development and instead are provided with the intention to entertain or distract [31, 61, 79]. Future studies should seek to observe the social components of recreational programs that contribute a sense of coherence, value and purpose for residents. This includes standardising activity participation reporting and measurement to include frequency, duration, level of social engagement as well as satisfaction and perceived social support within long-term care to gather a more holistic view of residents’ social context that accounts for the interaction of life experience, life stage and the environment [80]. Emerging research from population-based studies suggests that the role of risk factors for cognitive decline, including social integration and engagement amongst others, differ according to context (e.g., built environment, residential location) and life stage which may have ramifications for levels of cognitive preservation [25, 81]. Thus, a more extensive evaluation of the social context in long-term care will help to determine whether residents’ social needs are being met as well as explore the mechanisms within these psychosocial and recreational services that are associated with resident cognition.

## Conclusion

In conclusion, our current study advances the understanding of the relationship between social networks and cognitive function in the context of long-term care. We suggest that a larger social network may be associated with cognitive health benefits for older adults accessing long-term care services. However, despite this potential, the persistent challenge of fostering social inclusion through conventional psychosocial service delivery remains evident for policymakers and long-term care service providers. Thus, there is an evolving realisation that traditional or customary approaches may not yield the presumed efficacy in promoting both social and cognitive wellbeing. Consequently, there is a need for further investigation into the quality of social interactions and the interpersonal dynamics within the social context that underlie the design and implementation of service provision. Such research endeavours will deepen our comprehension of how social inclusion strategies impact cognitive health, providing valuable insights for the improvement of long-term care practices.

## Data Availability

The datasets used and/or analysed during the current study are available from the corresponding author on reasonable request.

## Abbreviations

LSNS-12: Lubben Social Network Scale
EQ5D5L: EuroQol 5D
MoCA: Montreal Cognitive Assessment
